# Genome-Wide Identification and Expression Analysis of *Hsp70* Gene Family of *Procambarus clarkii* Reveals Its Immune Role in Response to Bacterial Challenge After Non-Lethal Heat Shock

**DOI:** 10.3390/ani15142150

**Published:** 2025-07-21

**Authors:** Xin Zhang, Xiuhong Cai, Shirui Yue, Zhangxuan Chen, Yulong Sun, Lei Cheng, Yewen Xi, Shunchang Wang

**Affiliations:** 1 School of Biological Engineering, Huainan Normal University, Huainan 232001, China; 2 Anhui Huaihe River Basin Center for Aquatic Animal Epidemic Disease Protection and Control, Huainan 232038, China; 3 Department of Aquaculture, Zhejiang Ocean University, Zhoushan 316022, China; 4 Anhui Provincial General Station of Aquatic Technology Extension, Hefei 230091, China

**Keywords:** non-lethal heat shock, *Hsp70* gene family, immune regulation, *Procambarus clarkii*

## Abstract

Water temperature significantly affects the physiological balance of aquatic organisms like crustaceans, and heat shock proteins (HSPs) are crucial for stress resistance and pathogen defense. This study conducted a genome-wide analysis to explore the functional characteristics of the *Hsp70* gene family in *Procambarus clarkii*. Fifteen *Hsp70* family members were identified, with several genes showing upregulation under non-lethal heat shock (NLHS) and pathogen challenges. RNA-Seq and qPCR analyses confirmed increased expression of certain *PcHsp70s* during NLHS, indicating NLHS activation of the *Hsp70* family to enhance immune regulation. dsRNA-mediated silencing of *Hsp70* led to downregulation of TLR pathway genes (e.g., *TLR1*, *TLR6*), suggesting *Hsp70* regulates the TLR signaling pathway for immune responses. These findings reveal that NLHS-induced *Hsp70* upregulation improves pathogen resistance, offering insights for addressing temperature fluctuations and disease outbreaks in aquaculture to optimize management practices.

## Simple Summary

Crayfish (*Procambarus clarkii*) face challenges from changing water temperatures and bacterial infections, which threaten aquaculture productivity. This study investigated how crayfish use “heat shock proteins” (HSPs), natural cell protectors, to withstand stress. We identified fifteen *Hsp70* genes that activate when crayfish experience mild heat stress or bacterial exposure. Experiments showed that these genes work with the immune system’s “alarm signals” (similar to toll-like receptors) to fight pathogens. When we reduced *Hsp70* activity, the crayfish’s immune response weakened, confirming their role in defense. Crucially, mild heat stress primed crayfish to better resist infections, suggesting water temperature management can boost their disease resistance. These findings offer aquaculture farmers a practical way to reduce crayfish illnesses, improving survival rates and supporting sustainable farming.

## 1. Introduction

Water temperature, as a pivotal abiotic regulatory factor, plays a fundamental role in sustaining the physiological equilibrium of aquatic organisms [1]. Extensive research has demonstrated that significant deviations from the species-specific optimal water temperature can severely compromise the immune defense mechanisms of crustaceans against pathogenic microorganisms, including bacteria and viruses [1,2]. This underscores the critical importance of water temperature regulation in preserving crustacean health. Amid the alarming advance of global warming, the resultant fluctuations in ambient temperatures have emerged as a direct and profound ecological stressor, affecting the metabolic processes, growth, and reproductive performance of crustaceans [3,4]. Notably, the frequent occurrence of extreme heat events has been shown to disrupt the physiological equilibrium of crustaceans, such as shrimp, precipitating a cascade of adverse effects that can culminate in individual mortality [5]. However, studies have shown that NLHS can act as a stimulus to trigger adaptive responses in organisms, thereby improving immune function [6,7,8].

Through long-term evolution, organisms have developed intricate and sophisticated regulatory networks to cope with various environmental stresses, including extreme temperatures and ultraviolet radiation [9,10]. Among these regulatory components, Heat shock proteins (HSPs) serve as key molecular chaperones, playing an essential role in the stress response of organisms [11,12]. HSPs swiftly respond to external stressors by being highly expressed, stabilizing the intracellular environment, and safeguarding protein functions, thereby significantly enhancing the organism’s stress resistance [13,14]. In particular, NLHS-induced upregulation of *Hsp70* family members has been demonstrated to augment the host’s pathogen resistance [6,7].

HSPs are classified into various families based on their molecular weights, including the HSP110, HSP90, HSP70, HSP60, HSP40, HSP10, and the small HSPs [15,16]. HSP70, one of the most ubiquitously present and highly conserved members of the HSP family, is found in the genomes of nearly all organisms [17,18,19]. Initially identified in *Drosophila melanogaster*, HSP70 has garnered considerable attention for its vital contributions to abiotic stress, regulation of growth and development, and disease resistance [20]. The HSP70 protein family is distinguished by its highly conserved sequences and distinctive structural characteristics [21]. The primary function of HSP70 is to assist in the proper folding of intracellular peptide chains, rendering it a central molecular chaperone in protein stabilization, folding, and transport [22,23]. Its widespread distribution and robust conservation underscore its critical role across various organisms and cell types [24]. In cellular tolerance mechanisms, HSP70 effectively maintains cellular homeostasis by inhibiting the generation of harmful factors such as reactive oxygen species and modulating death receptor signaling pathways [12,25]. Moreover, HSP70 is intricately involved in regulating apoptosis and autophagy, demonstrating its anti-apoptotic functions, which are essential for cell survival and proliferation [26,27].

The red swamp crayfish (*Procambarus clarkii*), indigenous to the southern United States and northern Mexico, was introduced to China from Japan in the 1930s [28]. It has since become the most extensively farmed freshwater crayfish species in the country [29]. In 2023, *P. clarkii* production in China reached 3.16 million tons, ranking fourth among freshwater aquaculture species [30]. However, the rapid expansion of the *P. clarkii* aquaculture industry has been accompanied by frequent disease outbreaks, leading to substantial economic losses [31]. As an ectothermic species, *P. clarkii* can actively adjust its physiological activities in response to rapid fluctuations and seasonal changes in environmental temperature. However, when water temperatures exceed the species’ tolerable range, the accumulation of toxic reactive oxygen species (ROS) intensifies, damaging various cellular components and potentially leading to mortality [8,32]. In contrast, NLHS treatments exert the opposite effect; this “fever” process not only averts harm but also enhances immune function, thereby increasing the organism’s tolerance to pathogens [32,33].

With the advancement of high-throughput sequencing technologies and the accumulation of genomic and transcriptomic data, significant progress has been made in the comprehensive genomic analysis of the *Hsp70* gene family in aquatic animals. The number of *Hsp70* gene family members and their capacity to respond to various environmental factors have been extensively studied in species such as *Oncorhynchus mykiss* [34], *Larimichthys crocea* [35], *Apostichopus japonicus* [36], *Portunus trituberculatus* [17], and *Monodonta labio* [20]. For instance, 16 *Hsp70* genes have been identified in *O. mykiss* [34], while 17 *Hsp70* genes were discovered in the genome of *L. crocea* [35]. The *A. japonicus* genome harbors 15 *Hsp70* genes [36], 9 *Hsp70* gene family members were identified in *P. trituberculatus* [17], and 15 *Hsp70* members were found in *M. labio* [20]. These *Hsp70* genes play a crucial role in the organism’s response to environmental changes.

To further investigate the function of *Hsp70* gene family members, a genome-wide identification of *Hsp70* genes in *P. clarkii* was performed, and their gene expression levels under temperature stress and pathogenic infection were examined. This study not only helps clarify the evolutionary relationship and regulatory mechanisms of *Hsp70* family members in responding to environmental changes in crustaceans, but also offer valuable insights into how NLHS induces *Hsp70* genes expression to enhance the body’s immune regulatory capacity. The findings will serve as essential resources for functional research and aquaculture practices involving economically important crustacean species.

## 2. Materials and Methods

### 2.1. Ethics Statement

All of the study design and animal experiments were conducted following the guidelines of Animal Care and Use Committee of Huainan Normal University.

### 2.2. Genome-Wide Identification and Protein Characteristic Analysis of the Hsp70 Gene Family

The genome and annotation data of *P. clarkii* (GCA_040958095.1) were obtained from the NCBI database (https://www.ncbi.nlm.nih.gov/) (accessed on 8 October 2024). In the Pfam database (https://www.ebi.ac.uk/interpro/entry/pfam/) (accessed on 10 October 2024), the Hidden Markov Model seed file corresponding to the HSP70 domain (PF00012) was identified and used as a query to search for candidate sequences containing the HSP70 domain within the annotated protein-coding genes of the crayfish genome using HMMER software (v3.2; http://hmmer.org/) (accessed on 15 October 2024). Simultaneously, the *Hsp70* sequence of *Eriocheir sinensis* was downloaded from NCBI and used as a query sequence for BLASTP with an e-value of 1 × 10^−5^. Based on these results, potential members of the *Hsp70* family in *P. clarkii* were identified by taking the intersection of the HMMER and BLASTP results for subsequent analyses. Subsequently, public databases such as CDD (https://www.ncbi.nlm.nih.gov/Structure/bwrpsb/bwrpsb.cgi) (accessed on 17 October 2024) and SMART (http://smart.embl-heidelberg.de/) (accessed on 17 October 2024) were used to confirm the presence of HSP70 characteristic sequences. Protein sequences lacking these characteristic domains were excluded, leading to the final identification of the HSP70 family members in *P. clarkii*. The amino acid number, molecular weight (MW), and isoelectric point (pI) of the *Hsp70* genes were analyzed using the ExPASy ProtParam tool (http://web.expasy.org/protparam/) (accessed on 20 October 2024).

### 2.3. Gene Structure, Protein Domain, and 3-Dimensional Structural Analysis of P. clarkii Hsp70

The locations of exons and introns in the *Hsp70* genes were determined from the gff3 file of the *P. clarkii* genome annotation and extracted using TBtools software (version 2.310) [37]. To investigate the structural characteristics of the *P. clarkii* HSP70 proteins, conserved motifs were analyzed using the MEME online tool (https://meme-suite.org/meme/) (accessed on 22 October 2024), with the maximum number of motifs set to 10. The results were then visualized using TBtools software. The protein sequences were submitted to the SOPMA online tool for secondary structure prediction. The tertiary structure of HSP70 was predicted using the Swiss-Model online tool; only models with a similarity between the template and target sequences greater than 30% were accepted.

### 2.4. Chromosome Localization, Gene Duplication, and Phylogenetic Analysis of PcHsp70 Family

The genome annotation file of *P. clarkii* was retrieved from its genome database, and the chromosomal locations of the *Hsp70* family members, as well as the chromosome lengths, were extracted using the Gene Location Visualize from the GTF/GFF plugin in TBtools software. A chromosomal location map of the *P. clarkii Hsp70* genes was then generated. The collinearity of *P. clarkii* and *E. sinensis* was examined through the utilization of MCScanX [38]. Protein sequences derived from both species were aligned with the BlastP program. The e-value was set at 1 × 10^−10^, and the max_target_seqs parameter was set to 5. The generated alignment files, in conjunction with the merged genome annotation files of the two species in gff3 format, were subsequently processed by MCScanX. This analysis was instrumental in the identification of segmental and tandem duplication genes. Multiple sequence alignment was performed using Bioedit software (version 7.0.9). A phylogenetic tree was then constructed with the Maximum-Likelihood (ML) method in MEGA 6.0 software, employing a bootstrap value of 500 and default settings for other parameters.

### 2.5. Animals and Sample Collection

*P. clarkii* (15–20 g each) required for the experiment were purchased from an aquaculture farm in Huainan City, Anhui Province. A total of 300 crayfish were used in this study. The crayfish were acclimated in 100 L rectangular tanks (50 crayfish per tank) with the culture system maintaining specific water parameters: temperature at 25 ± 1 °C, pH at 7.2 ± 0.4, ammonia nitrogen below 0.05 mg/L, and dissolved oxygen above 5.0 mg/L. They were fed once daily on a regular schedule with commercially available *P. clarkii* feed from a farm, and the culture system was periodically cleaned with 30% water changes every 2 days. After a 7-day acclimation period, during which the crayfish’s overall condition stabilized, they were used for subsequent experiments. The experiment was divided into two stages: the heat shock treatment stage (HSS) and the pathogen infection stage (PIS). In the HSS, the crayfish were evenly divided into two groups (130 crayfish per group) with the following treatments:

(1) Control group (NT_group): Crayfish were maintained in 26 °C water for 6 h without heat shock treatment.
(2) Heat shock treatment group (HT_group): Crayfish were subjected to heat treatment in a 32 °C water bath for 2 h, followed by recovery in 26 °C water for 4 h.

During the PIS, crayfish from both the NT_group and HT_group were further divided into two subgroups (60 crayfish per subgroup) for a *Vibrio parahaemolyticus* injection challenge, with the following treatments:

(1) HTC_group: The HT_group was injected with 30 μL of normal saline (NS).
(2) HTV_group: The HT_group was injected with 30 μL of *V. parahaemolyticus* (1.0 × 10^8^ CFU/mL).
(3) NTC_group: The NT_group was injected with 30 μL of NS.
(4) NTV_group: The NT_group was injected with 30 μL of *V. parahaemolyticus* (1.0 × 10^8^ CFU/mL).

The dosage of 1.0 × 10^8^ CFU/mL for *V. parahaemolyticus* was selected based on preliminary experiments, as it induces a significant immune response in *P. clarkii* without immediate mass mortality, facilitating observation of survival and gene expression changes.

Hemolymph was collected from six crayfish in each group of the HSS and PIS, at 3 h, 12 h, 24 h, and 48 h. The hemolymph was centrifuged at 2000× *g* for 10 min at 4 °C, and the supernatant was discarded. Hemocytes were isolated and immediately frozen in liquid nitrogen for RNA isolation.

### 2.6. Survival Rate Statistics

**Figure 1:**
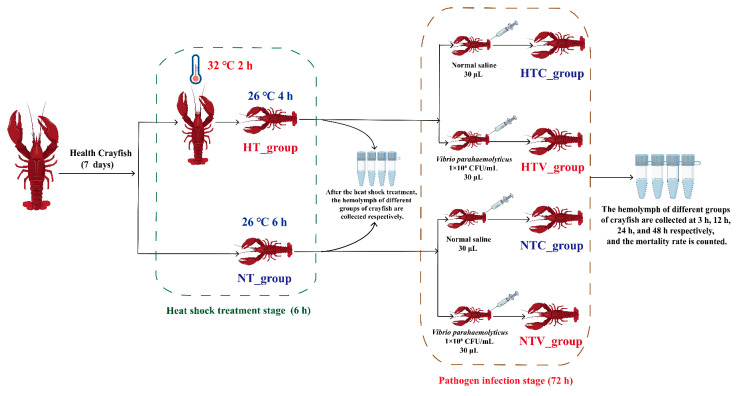
Experimental design of *P. clarkii* post-injection with *V. parahaemolyticus* following NLHS.

The survival rate of crayfish was recorded at 0 h, 3 h, 12 h, 24 h, and 48 h post-injection with *V. parahaemolyticus* following NLHS, and any dead crayfish were promptly removed from the tanks. GraphPad Prism version 8.0 was employed to conduct a log-rank test on the Kaplan–Meier survival curves, aiming to analyze the statistical discrepancies among different treatments. The details of the experimental setup are depicted in Figure 1.

### 2.7. The Expression Analysis of Hsp70 Genes in Response to Bacterial Challenge After NLHS

Total RNA was extracted from hemocytes using TRIzol reagent (Invitrogen, Carlsbad, CA, USA) following the manufacturer’s instructions. RNA quality was assessed using a NanoDrop 2000 spectrophotometer (Thermo Fisher Scientific, Waltham, MA, USA) and confirmed by electrophoresis. Subsequently, 1 µg of RNA was used for cDNA synthesis with M-MLV reverse transcriptase (Promega, Madison, WI, USA). Quantitative real-time PCR was performed to measure gene expression, using gene-specific primers (Table S1) and *Gapdh* (AB094145) as the housekeeping gene. The reaction was conducted in a LightCycler 96 system with 10× SYBR Green Master Mix, diluted cDNA, and primers, following cycling conditions: 95 °C for 1 min, 40 cycles of 95 °C for 15 s and 60 °C for 1 min. Melting curve analysis ensured primer specificity. Relative expression was calculated using the ΔΔCT method, with triplicate tests per sample. Statistical analysis was performed via *t*-test (*p* < 0.05) in SPSS 20, and a heatmap was generated in R v4.5.0. Additionally, RNA-Seq data (PRJNA1107630) generated from our own transcriptome sequencing of identical treatments were analyzed to supplement the expression profiles of *Hsp70* family members.

### 2.8. Suppression of Hsp70 Gene Expression by Double-Stranded RNA (dsRNA)

Based on the expression correlation analysis, the transcription level of *Hsp70* (LOC123759427) was significantly associated with the TLR signaling pathway, indicating its potential regulatory role in immune responses. To further investigate the functional interaction between *Hsp70* and the TLR pathway, RNA interference (RNAi) was performed to knockdown *Hsp70* expression. The green fluorescent protein (GFP) gene from pEGFP-N1 served as a negative control, with primer sequences for *Hsp70* and *GFP* listed in Table S1. PCR-amplified *Hsp70* and *GFP* fragments were purified, sequenced, and used as templates for single-stranded RNA (ssRNA) synthesis with T7 RNA polymerase (Promega). Sense and antisense ssRNAs were annealed at 75 °C for 15 min, 65 °C for 15 min, and cooled to room temperature at 0.1 °C/s to generate dsRNA. *Hsp70* dsRNA (5 μg/mL) was directly added to hemocyte culture medium without transfection reagents, with *GFP* dsRNA as the control [39]. A medium-only group served as the blank control. Each treatment included six biological replicates, incubated at 27 °C for 6 h, 12 h, and 24 h. Hemocytes were harvested for qRT-PCR analysis, using *Gapdh* as the reference gene to quantify *Hsp70* expression levels after knockdown and downstream TLR pathway gene expression.

## 3. Results

### 3.1. Survival Rate of P. clarkia Post Injection with V. parahaemolyticus Following NLHS

**Figure 2:**
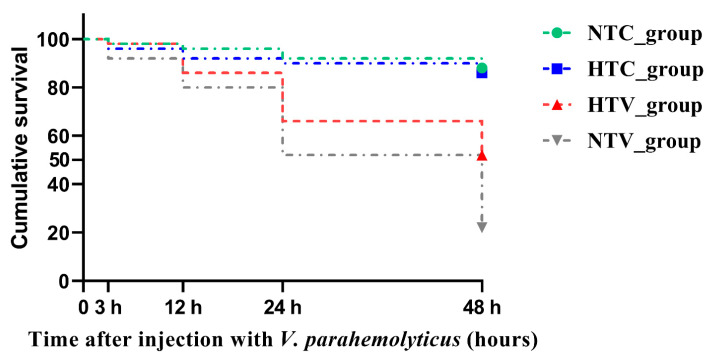
Cumulative survival using the Kaplan–Meier method of *P. clarkii* post-injection with *V. parahaemolyticus* following NLHS. “Cumulative survival” represents the proportion of crayfish that remained alive from the start of the experiment (0 h) up to each specified time point (3 h, 12 h, 24 h, 48 h). It was calculated as (number of surviving crayfish at each time point / initial number of crayfish in the group) × 100%. The time started after NLHS is denoted by “0–48 h”. This survival experiment was repeated three times. The meanings of group abbreviations are as follows: (1) HTC group: The HT_group was injected with 30 μL of normal saline (NS); (2) HTV_group: The HT_group was injected with 30 μL of *V. parahaemolyticus* (1.0 × 10^8^ CFU/mL); (3) NTC_group: The NT_group was injected with 30 μL of NS; (4) NTV_group: The NT_group was injected with 30 μL of *V. parahaemolyticus* (1.0 × 10^8^ CFU/mL).

In order to evaluate the effect of NLHS on the resistance of *P. clarkii* to pathogen infection, the surviving numbers of *P. clarkii* were enumerated. The log-rank test demonstrated that, over time after injection with *V. parahaemolyticus*, the mortality rates of both the HTV_group and the NTV_group increased. However, the mortality rate of the HTV_group remained lower than that of the NTV_group. This indicates that NLHS provides a certain degree of protective effect against *V. parahaemolyticus* infection, as evidenced by the relatively better survival performance of the HTV group compared to the NTV group (Figure 2).

### 3.2. Identification and Characterization of Hsp70 Genes in P. clarkii

A total of fifteen *Hsp70* genes were identified in *P. clarkii* through genome-wide analysis. These genes were meticulously named based on their unique characteristics or chromosomal locations, facilitating clear differentiation and further study (Table 1). The encoded proteins ranged from 517 to 696 amino acids in length, which led to differences in protein sizes. This size variation can influence the proteins’ functions, as larger proteins may have more complex structures and potentially more diverse roles. The molecular weights of the proteins fell within the range of 55.1–78.1 kDa, reflecting the mass differences resulting from the varying amino acid sequences. The theoretical pI values, which ranged between 5.27 and 5.98, indicated the isoelectric points of the proteins. These values are crucial as they can affect the proteins’ charge and solubility under different pH conditions, potentially influencing their interactions with other molecules in the cell (Table 1).

### 3.3. Multiple Sequence Alignment and Phylogenetic Analysis of PcHSP70s

**Figure 3:**
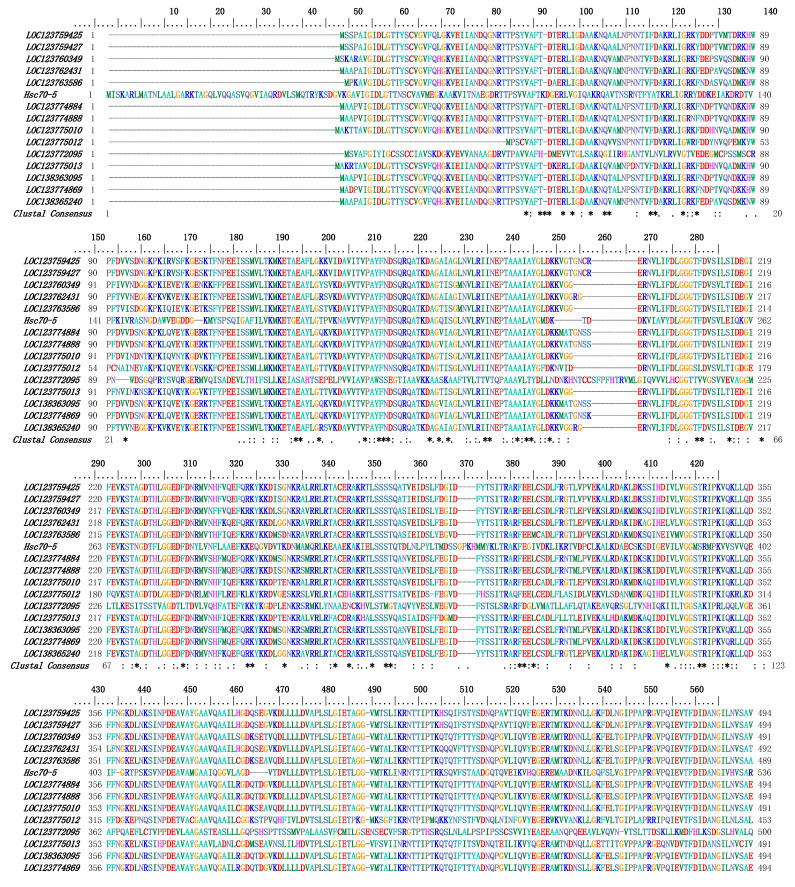

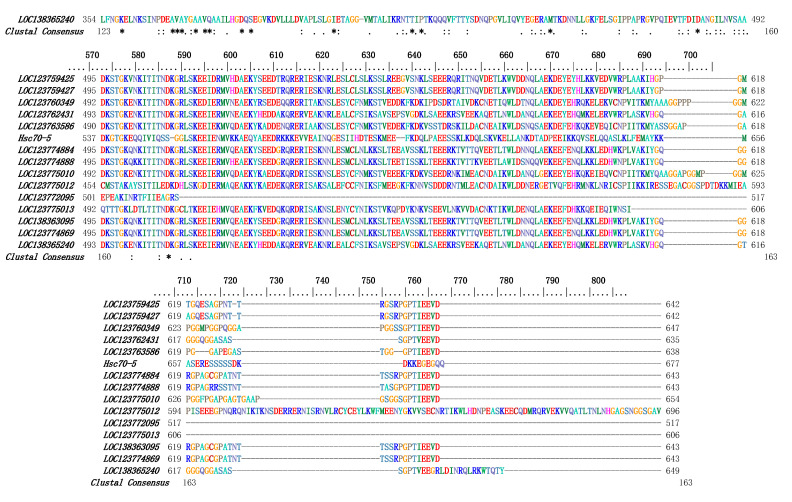
The multiple sequence alignment result of *Pc*HSP70 proteins. The multiple sequence alignment result of PcHSP70 proteins. The asterisks (*) indicate positions that have a single, fully conserved residue across all sequences in the alignment; the co-lons (:) represent positions that have conservation between groups of strongly similar properties; the periods (.) denote positions that have conservation between groups of weakly similar proper-ties.

**Figure 4:**
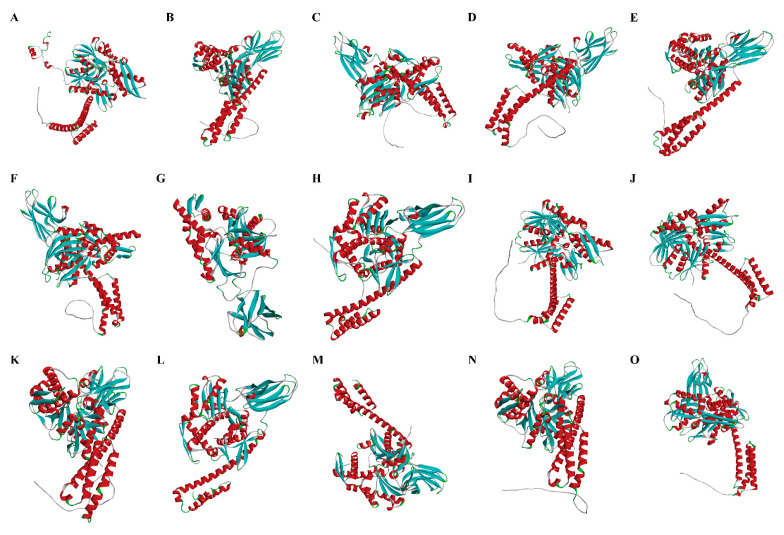
The predicted 3D structure of *Pc*HSP70s. (**A**–**O**) The 3D-structure of Hsc70-5, LOC123759425, LOC123759427, LOC123760349, LOC123762431, LOC123763586, LOC123772095, LOC123774869, LOC123774884, LOC123774888, LOC123775010, LOC123775012, LOC123775013, LOC138363095, LOC138365240, respectively.

To gain a deeper understanding of the similarities among *Pc*HSP70s, multiple-sequence alignment analysis was carried out using Bioedit software with default parameters. The outcome demonstrated that specific domains within *Pc*HSP70s, such as the HSP70 domain, were relatively conserved. However, the identity of the amino-acid sequences outside these conserved domains was remarkably low. In essence, although all these genes belong to the *Hsp70* gene family, significant differences exist in their amino-acid sequences (Figure 3). The three-dimensional structures of 15 members of the *Pc*HSP70 family were constructed by using SWISS-Model. A common globular core was possessed by all of them, which was likely crucial for chaperone activity. However, they also exhibited differences in folding patterns. Their functional specialization in substrate recognition and stress response may be underlain by these different folding patterns (Figure 4).

**Figure 5:**
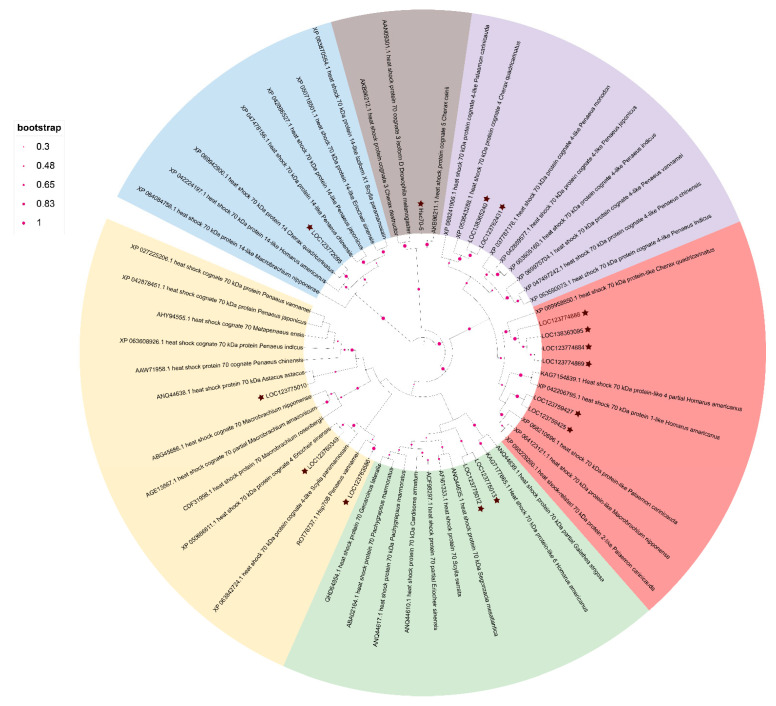
Phylogenetic analysis of *Pc*HSP70s from selected organisms. A phylogenetic tree was constructed by MEGA6 with the Neighbor Joining method and a bootstrap of 500 replications. Percent bootstrap values (500 bootstrap replications) are indicated in every branch. The stars represent different family members of Hsp70 in *P. clarkii*.

The phylogenetic analysis yielded an evolutionary tree primarily categorized into six major clades: HSP70, HSP70-like, HSP70-14, HSC70, and HSC70-4 (Figure 5). *Pc*HSP70 sequences were distributed across these clades. Some *Pc*HSP70s clustered closely with members of the HSP70 clade, indicating a close evolutionary relationship and potentially conserved functions related to stress responses. Others grouped with the HSC70 clade, suggesting roles in normal cellular protein-folding processes. The phylogenetic tree classifies *Pc*HSP70s into two major categories: classical stress-responsive HSP70s and constitutively expressed HSC70s involved in protein folding. This divergence implies functional specialization in stress adaptation and basal cellular maintenance (Figure 5).

### 3.4. Conserved Motifs, CDD Domain, and Gene Structure of Hsp70 Genes

**Figure 6:**
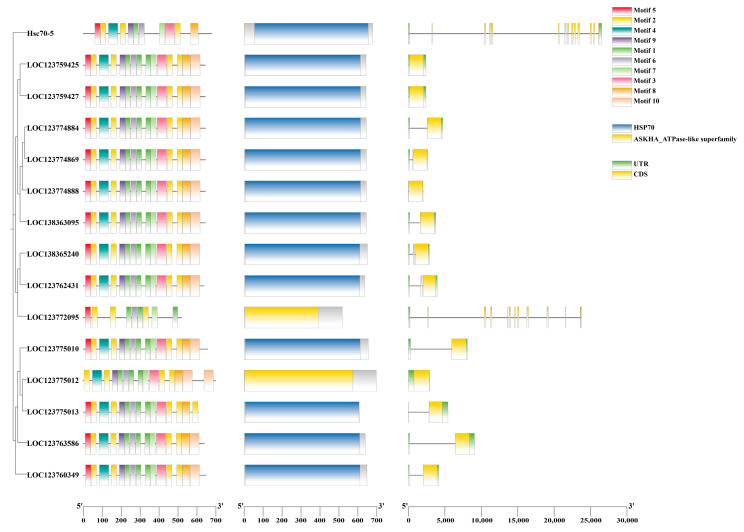
Protein domains and gene structures of the *Pc*HSP70s family. Different motifs and domains are displayed using colorful bars. Exons and 5′ UTR/3′ UTR are displayed using yellow bars and green bars. Black lines denote introns.

The motif analysis of *Pc*HSP70 proteins, as illustrated in Figure 6, revealed a conserved HSP70 domain across all family members, which is critical for their chaperone activity in protein folding and stress response. This core domain underscores the common functional mechanism shared by these proteins, enabling them to bind unfolded or misfolded proteins and facilitate proper folding. Notably, while the majority of motifs were associated with the canonical HSP70 domain, there were variations in non-core motifs among different family members. For instance, two *Pc*HSP70 proteins harbored an additional ASKHA_ATPase-like domain, which functions as a conformational hydrolase (e.g., in HSP70-mediated ATP hydrolysis) or a metabolite kinase (e.g., catalyzing phosphoryl group transfer from ATP) (Figure 6).

### 3.5. Chromosomal Location and Gene Duplication Analysis of Hsp70 Genes

**Figure 7:**
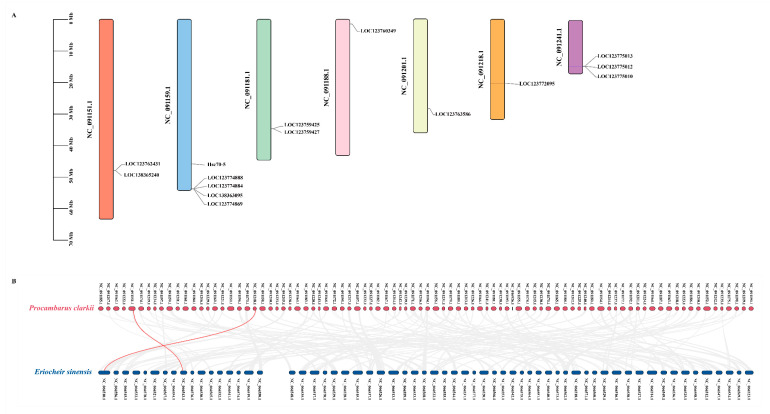
Chromosome distribution and gene collinearity analysis of *PcHsp70s*: (**A**) the diagram of chromosome distribution of *PcHsp70s*; (**B**) gene collinearity analysis of *PcHsp70s* with those from the *E. sinensis* genomes. Purple and red short sticks represent scaffolds from the *P. clarkii* and *E. sinensis*, respectively. The red lines indicate *Hsp70* gene pairs between species, and the gray lines represent other gene pairs with collinear relationships.

The chromosomal distribution of *PcHsp70* genes was analyzed, and the results revealed that fifteen *PcHsp70* genes were irregularly distributed on seven chromosomes (Figure 7). Most of these genes were randomly scattered at different positions on the chromosomes. Chromosome NC_091159.1 stood out as it contained the highest number of *Hsp70* genes, with a total of five (*Hsc70-5*, *LOC123774888*, *LOC123774884*, *LOC138363095*, and *LOC123774869*). Following this, NC_091241.1 harbored three members (*LOC123775013*, *LOC123775012*, and *LOC123775010*), indicating that different chromosomes contribute differently to the *PcHsp70* gene family (Figure 7A).

To explore the gene duplication events within the *PcHsp70* family, we analyzed the collinearity relationships between *P. clarkia* and *E. sinensis*. A total of two pairs of tandem duplication genes were identified. For instance, *LOC123762431* were tandemly duplicated and located on NC_091151.1. The close proximity of these tandemly duplicated genes on the chromosome suggests that they may have originated from a recent duplication event. Another example is *LOC123772095*, which were found on NC_091218.1. These tandem duplication events are likely to have contributed significantly to the expansion of the *Hsp70* gene family in *P. clarkia* (Figure 7B).

### 3.6. Expression Patterns of PcHsp70 Genes in Different Tissues

**Figure 8:**
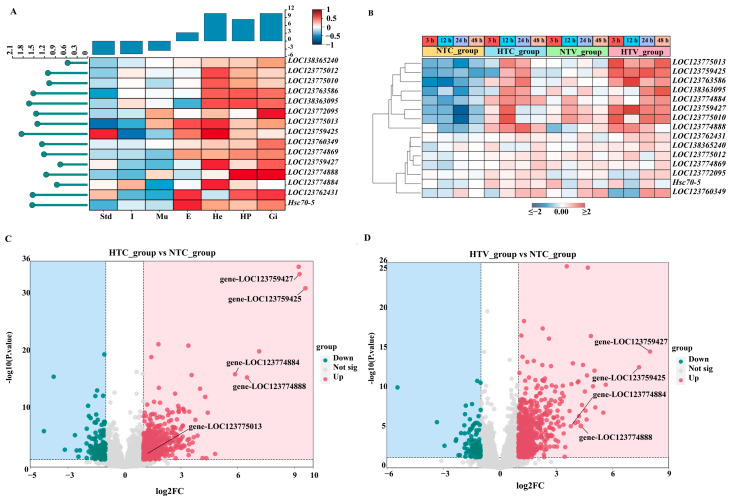
The expression profiles of *PcHsp70* genes: (**A**) the expression level of *PcHsp70* genes in hemocytes (He), stomach (Std), intestine (I), hepatopancreas (Hp), eyestalk (E), gill (Gi), and muscle (Mu); (**B**) the expression level of *PcHsp70* genes in response to *V. parahaemolyticus* following NLHS; (**C**) volcano plot of *Hsp70* differentially expressed genes (DEGs) distribution trends between HTC_group vs. NTC_group; (**D**) volcano plot of *Hsp70* differentially expressed genes (DEGs) distribution trends between HTV_group vs. NTC_group. The significantly upregulated and down-regulated genes expressed, adjusted at default parameters as padjust <0.05, were represented as red and green dots, respectively.

The expression levels of 15 *PcHsp70* genes were quantified via qRT-PCR across seven tissues: hemocytes (He), stomach (Std), intestine (I), hepatopancreas (Hp), eyestalk (E), gill (Gi), and muscle (Mu). The results, as presented in Figure 7, indicated that the expression patterns of these *Hsp70* genes were significantly varied among different tissues. As shown, some genes, like *LOC123763586* and *LOC138363095,* were highly expressed in the hepatopancreas, while minimal expression was shown in the stomach or eyestalk, respectively. Multiple genes, including *LOC123775013*, *LOC123759427*, *LOC123759425*, *LOC123774884*, *LOC123775010,* and *LOC123775012*, were highly expressed in the hemocyte, but low expression was detected in the muscle, intestine, or stomach. There were also genes such as *LOC123762431* and *Hsc70-5* that were highly expressed in the eyestalk. In general, distinct tissue-specific expression patterns among the fifteen *Hsp70* genes in *P. clarkii* were demonstrated by these findings (Figure 8A).

### 3.7. Expression Patterns of Hsp70 Genes in Response to Bacterial Challenge After NLHS

The expression patterns of *PcHsp70* genes were investigated under NLHS treatment and *V. parahaemolyticus* infection. Under NLHS treatment, the expression levels of eight *Hsp70* genes, such as *LOC123774884*, *LOC123759427*, *LOC123759425*, *LOC123774888*, *LOC123775010*, *LOC123763586*, *LOC138363095*, and *LOC123775013*, were significantly upregulated (*p* < 0.05) (Figure 8B). Following the *V. parahaemolyticus* infection, changes in the expression levels of certain *Hsp70* genes were observed. In particular, *LOC123759427* and *LOC123759425* showed a significant increase in expression at different time points. However, not all members of the *Hsp70* gene family responded positively to these experimental conditions. The other seven members of the *Hsp70* gene family exhibited no significant expression changes across different treatment groups (Figure 8B).

RNA-Seq data from identical treatments were analyzed to supplement *Hsp70* family expression profiles. Volcano plots visualize differential expression in three comparisons: HTC_group vs. NTC_group, HTV_group vs. NTC_group, and NTV_group vs. NTC_group (Figure 8C,D). In both HTC and HTV comparisons, *PcHsp70* family genes, such as *LOC123759427*, *LOC123759425*, *LOC123774884*, and *LOC123774888*, showed significant upregulation (|log_2_FC| > 1, *p* < 0.05). For instance, *LOC123759427* and *LOC123759425* exhibited extreme upregulation (Figure 8C,D). In contrast, no significant changes in expression were detected for any *Hsp70* family members in the NTV_group vs. NTC_group comparison, indicating that non-heat shock Vibrio challenge alone does not induce *Hsp70* transcription. These RNA-Seq-derived expression patterns were consistent with qPCR validations, confirming robust induction of core *Hsp70* genes under combined heat shock and bacterial stresses (Figure 8A–D).

### 3.8. The Protein-Protein Interaction of HSP70s

The above results showed that the expression levels of certain *Hsp70* genes in the hemocytes of *P. clarkii* under NLHS treatment followed by *V. parahaemolyticus* infection were significantly changed. For example, *LOC123759427* and *LOC123759425* were notably upregulated in different phases, suggesting they might play crucial roles in the response to such stress and infection conditions. To gain a better understanding of its functions, the proteins that might interact with the protein encoded by HSPA1L (Heat Shock 70 KDa Protein 1-Like) and HSPA1B (Heat Shock 70 KDa Protein 1-B) were predicted using the STRING 12.0 software (https://cn.string-db.org/) (accessed on 10 November 2024). The result showed that the protein encoded by HSPA1L and HSPA1B could interact with several immune-related proteins, including TLR1, TLR2, TLR4, TLR6, and MyD88.

**Figure 9:**
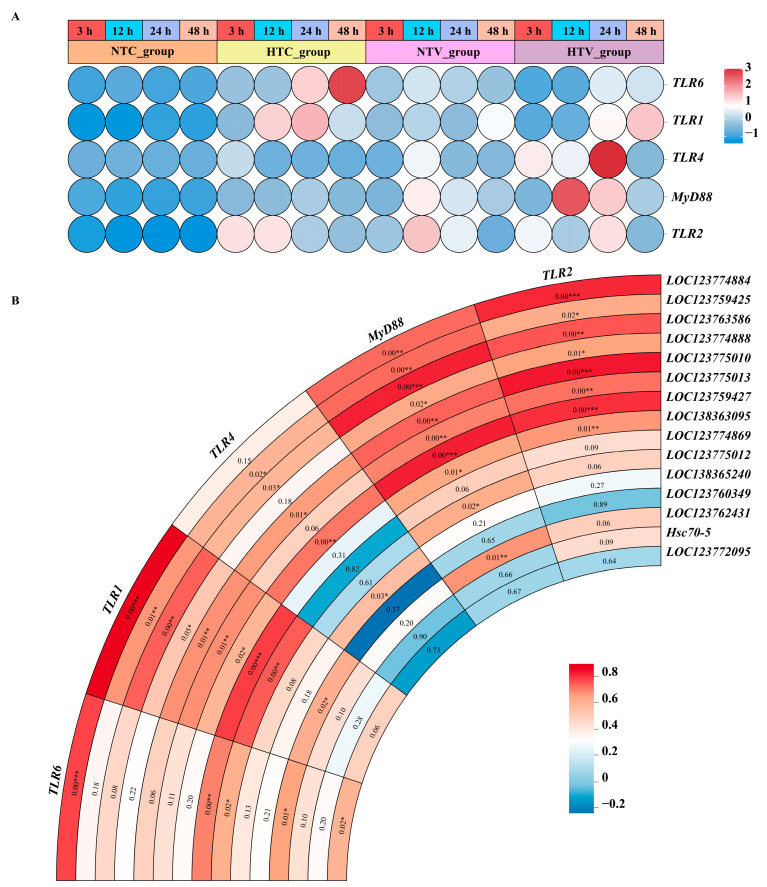
Analysis of *Pc*HSP70s and related immune gene expression: (**A**) the expression level of immune-related genes predicted to have interaction with *Hsp70s* in response to bacterial challenge after NLHS; (**B**) the correlation relationships between *PcHsp70s* and immune-related genes predicted to have interaction with them. Values marked on the blocks are correlation coefficients, and asterisks (*) indicate the level of statistical significance (e.g., * for *p* < 0.05, ** for *p* < 0.01, *** for *p* < 0.001).

To further explore the role of *Hsp70* genes in the immune response of *P. clarkii*, qPCR was used to analyze the expression changes of genes that the STRING database predicts may interact with *Hsp70* genes. As shown in Figure 9A, *TLR1*, *TLR2*, *TLR4*, *TLR6*, and *MyD88* were all upregulated at different times in the HTC_group or HTV_group. Meanwhile, a correlation heatmap was generated using the CNSknowall platform (https://cnsknowall.com/) (accessed on 22 November 2024). The results revealed a positive correlation between the expression levels of certain *Hsp70* genes and those of their putative interacting genes, suggesting potential co-regulatory relationships in the cellular response to stress. Especially, the expression of *TLR1*, *TLR2*, and *MyD88* showed significant correlation with that of *LOC123774884*, *LOC123759427*, *LOC123759425*, *LOC123774888*, *LOC123775010*, *LOC123763586*, *LOC138363095*, and *LOC123775013* (Figure 9B).

### 3.9. Effect of dsRNA Exposure Assay for Hsp70 Gene Expression

**Figure 10:**
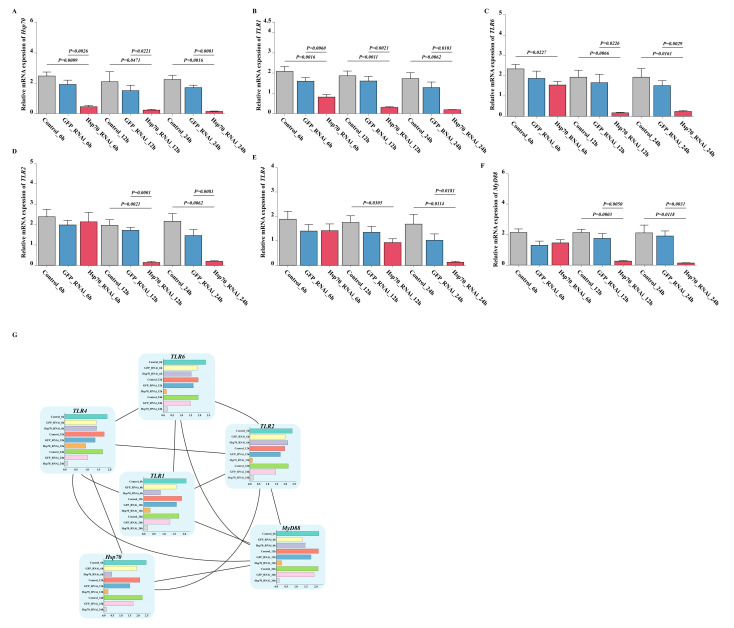
Expression analysis of *Hsp70* and related immune genes after *Hsp70* silencing by double-stranded RNA interference: (**A**–**F**) relative expression levels of (**A**) *Hsp70*, (**B**) *TLR1*, (**C**) *TLR2*, (**D**) *TLR4*, (**E**) *TLR6*, and (**F**) *Myd88* following *Hsp70* knockdown; (**G**) the interaction network of *Hsp70* and TLR signal pathway-related genes after *Hsp70* was knockdown.

The expression of genes associated with the TLR signaling pathway in hemocytes after *Hsp70* silencing by dsRNA was assessed by qPCR. The results indicated that *Hsp70* gene expression in the experimental group was substantially lowered at all time points relative to the control group (*p* < 0.05) (Figure 10A). Similarly, other genes in the pathway: *TLR1*, *TLR2*, *TLR4*, *TLR6*, and *MyD88* demonstrated significant downregulation at different time points in the experimental group compared with the control group (*p* < 0.05) (Figure 10B–F). In particular, *TLR1* and *TLR6* showed robust interference effects and were persistently downregulated at each time point of the experiment (*p* < 0.05) (Figure 10B,C). Subsequently, the software was employed to visualize this interaction result (Figure 10G).

## 4. Discussion

HSPs, often referred to as molecular chaperones, play an essential role in helping proteins refold and preventing protein denaturation during stressful conditions. They are crucial for regulating cellular stress tolerance [19,40]. In this study, we set out to identify and screen the *PcHsp70* gene family on a genome-wide scale, focusing particularly on their roles in NLHS and subsequent immune responses to pathogen infections. The log-rank test results revealed that following the injection of *V. parahaemolyticus*, the mortality rates of both the HTV_group and the NTV_group steadily increased over time. However, the mortality rate of the HTV_group was consistently lower than that of the NTV_group. This finding strongly indicates that NLHS, the brief heat shock treatment, confers a significant degree of protective efficacy against *V. parahaemolyticus* infection. Our findings shed new light on the molecular mechanisms underlying stress adaptation and immunity in crustaceans, which have practical implications for the healthy culture of *P. clarkii*.

Through analysis, a total of fifteen *Hsp70* family genes were identified in the *P. clarkii* genome. The proteins encoded by these genes exhibit a range of physicochemical properties, including differences in length, molecular weight, and isoelectric point. This diversity suggested the potential for functional specialization among these proteins. For instance, the larger isoforms may possess complex domain structures that allow them to perform multiple roles in substrate recognition, protein folding, and stress signal transduction [41]. Multiple sequence alignment and phylogenetic analysis revealed a conserved HSP70 domain within *Pc*HSP70s, while significant variations in sequence consistency were noted in non-domain regions, which is consistent with the reported results [42]. Phylogenetic classification further divided *Pc*HSP70s into six distinct groups. Interestingly, some *Pc*HSP70s clustered with classic HSP70s linked to stress responses, while others resembled HSC70s involved in fundamental protein folding processes [43]. This suggests that *Pc*HSP70s might also have a dual role in managing environmental stress and maintaining intracellular homeostasis [25].

Chromosome mapping analysis highlighted that the 15 *Hsp70* genes were unevenly distributed across seven chromosomes, with NC_091159.1 having the highest gene density (five genes). This irregular distribution indicated that different genomic regions contribute variably to the amplification of the *Hsp70* family. Gene duplication, recognized as a key driver of evolutionary innovation, may facilitate the diversification of functional roles within this gene family [44]. Repetitive paralogs could acquire specialized functions related to stress adaptation or fine-tuning the regulation of protein folding [45]. Notably, this phenomenon was not exclusive to *P. clarkii*; similar amplification patterns have been observed in *L. crocea*, where the duplication of *Hsp70* genes has improved environmental adaptability [35]. Homology analysis revealed that homologous gene pairs (*LOC123762431*/*LOC127007335* and *LOC123772095*/*LOC126999721*) between *P. clarkii* and *E. sinensis* are conserved, underscoring their evolutionary stability and functional necessity since the divergence from common ancestors.

Tissue-specific expression profile analysis showed significant spatial differences in the expression of *Hsp70* genes across seven examined tissues (hemocytes, stomach, hepatopancreas, eyestalk, gills, and muscle). Significantly elevated expression levels were observed in metabolically active tissues, including the hepatopancreas, hemocytes, and gills. This finding was consistent with their critical roles in detoxification, immune surveillance, and environmental interaction [46,47]. As a vital digestive gland and immune organ, the hepatopancreas likely requires robust mechanisms to maintain protein homeostasis under both physiological and pathological conditions [48]. In contrast, the lower expression levels in the stomach, muscles, and intestines suggested that these tissues may rely on alternative chaperone systems or different stress-responsive pathways.

The dynamic regulation of the *Hsp70* gene family in *P. clarkii* following NLHS and *V. parahaemolyticus* infection provides essential insights into immune regulation. Our findings indicate that several *Hsp70* genes are significantly upregulated after NLHS, corroborated by RNA-Seq data and qPCR validation, thus activating the cell’s protective mechanisms [6,7]. Notably, LOC123759427 and LOC123759425 show extreme upregulation during pathogen challenges, emphasizing their dual roles in heat adaptation and anti-pathogen defense. The synergistic upregulation of these genes in response to both types of stress suggests a complex interaction between the stress response pathways and immune activation in *P. clarkii*, particularly as HSP70 may act as a core molecule regulating this intricate reaction [49]. Interestingly, no significant changes in *Hsp70* expression were observed in the non-thermal Vibrio challenge group (NTV_group vs. NTC_group), indicating that heat shock preconditions *Hsp70* activation. This phenomenon has also been documented in *Artemia franciscana*, where heat shock enhances pathogen resistance via HSP70 induction [6]. Similarly, NLHS may prime the immune system in *P. clarkii* by inducing *Hsp70*, thereby enhancing immune regulation against pathogen infections.

In comparison with other species, 17 *Hsp70* genes have been identified in *L. crocea*, six of which exhibit temperature-dependent regulation, signifying their critical role in responding to thermal stress [35]. This highlights a parallel with our findings in *P. clarkii* regarding the importance of *Hsp70* in adapting to environmental temperature changes. Furthermore, research on *Portunus trituberculatus* indicates that *Hsc70* and *HYOU1* homologous genes display different regulatory patterns under low-salinity stress, suggesting that various crustacean species adapt to distinct environmental stressors through unique gene regulatory mechanisms [17]. For instance, *A. franciscana* demonstrated a significant increase in resistance to pathogenic bacteria after a brief period of NLHS, exceeding a twofold elevation compared to control groups [6]. This finding supports the idea that NLHS could serve as an ecological immune priming strategy, similar to what we observe in *P. clarkii*, although the effectiveness of this strategy may vary based on each species’ physiological tolerance and recovery efficiency. Contrastingly, studies on *Litopenaeus vannamei* revealed that despite notable upregulation of *Hsp70* and immune-related proteins (e.g., phenoloxidase and hemocyanin) following NLHS, there was no corresponding increase in resistance to *V. harveyi* [7]. This discrepancy underscores the species-specific nature of stress–immune interactions and emphasizes the need to tailor stress preconditioning protocols for broader applicability and effectiveness. Furthermore, research on *M. labio* identified 15 *Hsp70* genes, revealing significant differential expression patterns concerning varying concentrations of nanoplastics (NPs), elucidating their pivotal role in stress adaptation [20]. This finding illustrates that, while thermal stress and environmental pollutants represent different types of stressors, the *Hsp70* gene family can effectively maintain protein homeostasis under various conditions. Consequently, this demonstrates the broad adaptability and evolutionary significance of *Hsp70* in responding to multiple environmental pressures, reinforcing its crucial role not only in *P. clarkii* but across diverse crustacean species.

Protein interaction network predictions suggest that proteins encoded by HSPA1L and HSPA1B may interact with key immune molecules in the TLR signaling pathway, including TLR1, TLR2, TLR4, TLR6, and MyD88. These interactions could enhance pathogen recognition through the activation of the TLR signaling pathway and downstream NF-κB [50]. Additionally, the observed positive correlation between *Hsp70* expression and putative interacting factors indicates a synergistic relationship in immune regulation. Our functional validation via dsRNA-mediated *Hsp70* silencing supports this hypothesis: silencing *Hsp70* results in significant downregulation of *TLR1*, *TLR6*, and *MyD88* at various time points. Cytoscape (v3.10.3) visualization further illustrates direct associations between *Hsp70* and TLR/MyD88 components, suggesting that HSP70 facilitates the proper folding of TLR proteins, which is crucial for efficient NF-κB activation during immune responses [51]. Specifically, *Hsp70* family genes may assist in the correct folding of proteins encoded by these interacting genes, optimizing their ability to recognize pathogens and ensuring normal function during immune responses [52,53]. This molecular interaction may enable *P. clarkii* to develop a stronger antibacterial defense, thereby improving its survival rate against pathogen infections. However, this study has limitations. It focused on specific NLHS conditions and a single pathogen, restricting generalization to other stress/pathogen scenarios. Hsp70-TLR pathway interactions were explored via gene expression; protein-level mechanisms need further study.

## 5. Conclusions

In conclusion, this study systematically identified 15 *Hsp70* genes in *P. clarkii* at the genome-wide level, revealing distinct structural features and chromosomal distributions. Phylogenetic analysis grouped these genes into six clades, indicating functional divergence between stress-inducible HSP70s and constitutive HSC70s. RNA-Seq data and qPCR validation showed that NLHS and *V. parahaemolyticus* infection synergistically upregulated *Hsp70s* in hemocytes, whereas no induction occurred under non-thermal Vibrio challenge alone. Functional assays confirmed that NLHS pretreatment enhanced survival against bacterial infection, correlating with HSP70 chaperone activity. dsRNA-mediated *Hsp70* silencing revealed concurrent downregulation of TLR pathway genes and Cytoscape-visualized interactions, suggesting *Hsp70* modulates innate immunity via TLR/MyD88 signaling. These findings establish a molecular framework for HSP70-mediated stress adaptation in crustaceans and highlight targets for aquaculture disease resistance improvement.

## Figures and Tables

**Table 1:** Basic information on *Hsp70* family members in *Procambarus clarki*.

Gene_Name	Gene_ID	Chromosome	Annotation	Number of Amino Acid	Molecular Weight	Theoretical pI	Instability Index	Aliphatic Index	Grand Average of Hydropathicity
LOC123759425	XM_045744490.2	NC_091181.1	heat shock 70 kDa protein 1-like	642	70,854.91	5.73	34.11	83.99	−0.469
LOC123759427	XM_045744492.2	NC_091181.1	heat shock 70 kDa protein 1	642	70,864.95	5.67	35	84.3	−0.462
LOC123760349	XM_045745963.2	NC_091188.1	heat shock 70 kDa protein cognate 4	647	71,168.32	5.28	35.85	77.6	−0.483
LOC123762431	XM_045748943.2	NC_091151.1	heat shock 70 kDa protein cognate 4	635	69,433.29	5.49	33.81	82.33	−0.457
LOC123763586	XM_045750772.2	NC_091201.1	heat shock cognate 71 kDa protein	638	69,882.92	5.23	37.94	77.7	−0.443
Hsc70-5	XM_045769046.2	NC_091159.1	heat shock 70 kDa protein cognate 5	677	73,443.6	5.73	32.52	84.11	−0.376
LOC123774884	XM_045769563.2	NC_091159.1	heat shock 70 kDa protein	643	71,026.16	5.41	38.04	80.39	−0.52
LOC123774888	XM_045769576.2	NC_091159.1	heat shock 70 kDa protein-like	643	71,043.14	5.6	37.71	81.29	−0.528
LOC123775010	XM_045769739.2	NC_091241.1	heat shock cognate 70 kDa protein	654	71,709.98	5.27	37.97	78.01	−0.467
LOC123775012	XM_045769740.2	NC_091241.1	heat shock cognate 71 kDa protein-like	696	78,070.67	5.98	38.32	79.44	−0.467
LOC123772095	XM_069311168.1	NC_091218.1	heat shock 70 kDa protein 14 isoform X1	517	55,113.17	5.89	44.58	94.08	0.145
LOC123775013	XM_069318964.1	NC_091241.1	heat shock cognate 71 kDa protein-like	606	67,565.77	5.51	35.67	87.84	−0.344
LOC138363095	XM_069322069.1	NC_091159.1	heat shock 70 kDa protein-like	643	71,054.21	5.41	37.4	80.39	−0.519
LOC123774869	XM_069322070.1	NC_091159.1	heat shock 70 kDa protein-like	643	71,139.28	5.41	37.14	80.23	−0.534
LOC138365240	XM_069325507.1	NC_091151.1	heat shock-related 70 kDa protein 2-like	649	71,249.39	5.79	33.51	81.91	−0.484

## Data Availability

The data presented in this study are available on request from the corresponding author.

## Supplementary Materials

The following supporting information can be downloaded at: https://www.mdpi.com/article/10.3390/ani15142150/s1; Table S1: All primer sequences in this study.
