# Supplementary material for: Genome-Wide Identification and Expression Analysis of Hsp70 Gene Family of Procambarus clarkii Reveals Its Immune Role in Response to Bacterial Challenge After Non-Lethal Heat Shock
Source: Animals (Basel). 2025 Jul 21;15(14):2150. doi: 10.3390/ani15142150 (PMC12291665; doi:10.3390/ani15142150)
Supplement: Supplementary file 1 [file animals-15-02150-s001.zip › animals-3738280-supplementary.pdf]

Table S1

Primers used in this article

Primer name	Nucleotide sequence (5'→3')	Purpose
*LOC123762431*_F	GCACGGCAAGGTGGAGAT	qRT-PCR
*LOC123762431*_R	TTGGGGTTCATGGCCACC	qRT-PCR
*LOC138365240*_F	GCACGGCAAGGTGGAGAT	qRT-PCR
*LOC138365240*_R	TTGGGGTTCATGGCCACC	qRT-PCR
*Hsc70-5*_F	GCACCAGGGGGAGAGAGA	qRT-PCR
*Hsc70-5*_R	CACGTGGTGCAGGTGGAA	qRT-PCR
*LOC123774884*_F	GGCCGTCTCCAGCAAACT	qRT-PCR
*LOC123774884*_R	TTCTCCGCCAGCTGGTTG	qRT-PCR
*LOC123774869*_F	GGCCGTCTCCAGCAAACT	qRT-PCR
*LOC123774869*_R	TTCTCCGCCAGCTGGTTG	qRT-PCR
*LOC123774888*_F	GGTGACCAGACTGACGGC	qRT-PCR
*LOC123774888*_R	CTGGGTCTGCTTGGTGGG	qRT-PCR
*LOC138363095*_F	CCCACCAAGCAGACCCAG	qRT-PCR
*LOC138363095*_R	TCATGGCTCGCTCTCCCT	qRT-PCR
*LOC123759427*_F	TCGAGCCAGGTTCGAGGA	qRT-PCR
*LOC123759427*_R	CACTTTGGGGATGCGGGT	qRT-PCR
*LOC123759425*_F	TCGAGCCAGGTTCGAGGA	qRT-PCR
*LOC123759425*_R	CACTTTGGGGATGCGGGT	qRT-PCR
*LOC123760349*_F	GCTCGACACGAACCAGCT	qRT-PCR
*LOC123760349*_R	GTCCTCCTGGCATACCGC	qRT-PCR
*LOC123763586*_F	CCACGTGGGGTTCCTCAG	qRT-PCR
*LOC123763586*_R	TTGCTGAGCCGTCCCTTG	qRT-PCR
*LOC123772095*_F	TGCGTGTAGTGGGCACAG	qRT-PCR
*LOC123772095*_R	TAACGCGGCTGACCACTG	qRT-PCR
*LOC123775010*_F	TGATTCTCAGCGCCAGGC	qRT-PCR
*LOC123775010*_R	ACATTGCGCTCACCACCA	qRT-PCR
*LOC123775013*_F	TGATTCTCAGCGCCAGGC	qRT-PCR
*LOC123775013*_R	ACATTGCGCTCACCACCA	qRT-PCR
*LOC123775012*_F	TCAAGCGCAACACACCCA	qRT-PCR
*LOC123775012*_R	AGCGCCCAAGAAGCTTGT	qRT-PCR
Gapdh-F	GCCCAGAACATCATCCCATCT	qRT-PCR
Gapdh-R	CGTCATCCTCAGTGTAACCCAAG	qRT-PCR
TLR1-F	GTTTCTCCACACCCCGGG	qRT-PCR
TLR1-R	AACGAGGTGCTCCAAGCC	qRT-PCR
TLR2-F	CACTCACAGCGCCAAGGA	qRT-PCR
TLR2-R	GTCACGATGGGCCACACA	qRT-PCR
TLR4-F	TCACACGCGATGAGGCTG	qRT-PCR
TLR4-R	AGAGTAGGAAGGCGGGCA	qRT-PCR
TLR6-F	ACAGCCGCATCAACGTGA	qRT-PCR
TLR6-R	AGTCATCAGCCCGTTGCC	qRT-PCR
MyD88-F	GAAACGCCTGCTGGACCT	qRT-PCR

MyD88-F	GCTCATGGGACTCGTGCA	qRT-PCR
Hsp70-RNAi-F	CAACGACAAGGGTCGTCTGA	RNAi
Hsp70-RNAi-R	TTGAGGGTTTCGTCCACCTG	RNAi
GFP-RNAi-F	GGATCCTAATACGACTCACTATAGGGATC	RNAi
GFP-RNAi-R	GGATCCTAATACGACTCACTATAGGGATC	RNAi
